# Retraction Note to: Failure of internal fixation of the anterior ring for unstable pelvic fractures, the experience of a single institute

**DOI:** 10.1186/s13018-022-03098-1

**Published:** 2022-04-02

**Authors:** Sheng Zhang, Huagui Mo, Yucheng Liu, Guohua Zhu, Bin Yu

**Affiliations:** 1 Department of Orthopedics, Nanfang Hospital, Southern Medical University, No. 1838, Guangzhou Avenue North, Baiyun District, Guangzhou, Guangdong Province China; 2 Department of Orthopedics, People’s Hospital of Hua Zhou, Huazhou, Guangdong Province China; 3 Department of Orthopedics, Jiangmen Central Hospital, Jiangmen, Guangdong Province China

## Retraction Note to: Orthop Surg Res (2021) 16:577 https://doi.org/10.1186/s13018-021-02735-5

The authors have retracted this article. After the publication, the authors engaged in follow up work which yielded different results to this article. The authors believe this is due to inconsistencies in the results of the surgeries and have therefore lost confidence in the reliability of the results.

Sheng Zhang, Huagui Mo, Yucheng Liu and Guohua Zhu agree to this retraction. Bin Yu has not responded to any correspondence from the publisher about this retraction.

